# Supplementary material for: Electrical Resistance Sensing of Epoxy Curing Using an Embedded Carbon Nanotube Yarn
Source: Sensors (Basel). 2020 Jun 5;20(11):3230. doi: 10.3390/s20113230 (PMC7309011; doi:10.3390/s20113230)
Supplement: Supplementary file 1 [file sensors-20-03230-s001.pdf]

sensors

MDPI

Article

Electrical Resistance Sensing of Epoxy Curing Using an Embedded Carbon Nanotube Yarn

Omar Rodríguez-Uicab [1,*], Jandro L. Abot [1] and Francis Avilés [2]

[1] Department of Mechanical Engineering, The Catholic University of America, Washington, DC 20064, USA; abot@cua.edu
[2] Centro de Investigación Científica de Yucatán A.C., Unidad de Materiales, Calle 43 No. 130 x 32 y 34 Col, Chuburna de Hidalgo, 97205 Mérida, Mexico; faviles@cicy.mx
* Correspondence: rodriguezuicab@cua.edu

Received: 1 May 2020; Accepted: 3 June 2020; Published: date

1. Supplementary Material

1.1. Rheometry of Epoxy Resins

Figure S1 shows the liquid viscosity of ER-A and ER-B epoxy resins listed in Table 1, as a function of temperature. The tests shown in Figure S1 were conducted on "part A" of the epoxy resin, i.e., the resin without curing agent added. The experiments were conducted using an ER 2000 rheometer of TA instruments, using a parallel plates accessory with 500 µm gap, with a heating rate of 5 °C/min from 30 °C to 150 °C, applying a 0.5 µN-m torque. To obtain reproducibility, three replicates of each polymer were analyzed. Figure S1a shows the variation of the viscosity of ER-A resin as a function of temperature. For this resin, it is seen that the viscosity quickly decreases from ~ 1625 cP at room temperature (RT, ~ 30 °C) to ~ 105 cP at 60 °C, and then levels off with only modest decrease in viscosity with increased temperature (T) for $T > 60$ °C. At the curing temperature (130 °C) the average viscosity of the three samples tested is only 59 cP. On the other hand, For ER-B (Figure S1b), the initial viscosity at RT is lower (953 cP) but the viscosity at the curing temperature (60 °C) is ~ 115 cP. Thus, even when at RT ER-A is more viscous than ER-B, at the corresponding curing temperature the viscosity of ER-A is almost half that of ER-B. The lower viscosity of ER-A at the curing temperature could promote more resin infiltration into the CNTY [1], as is discussed in the main text.

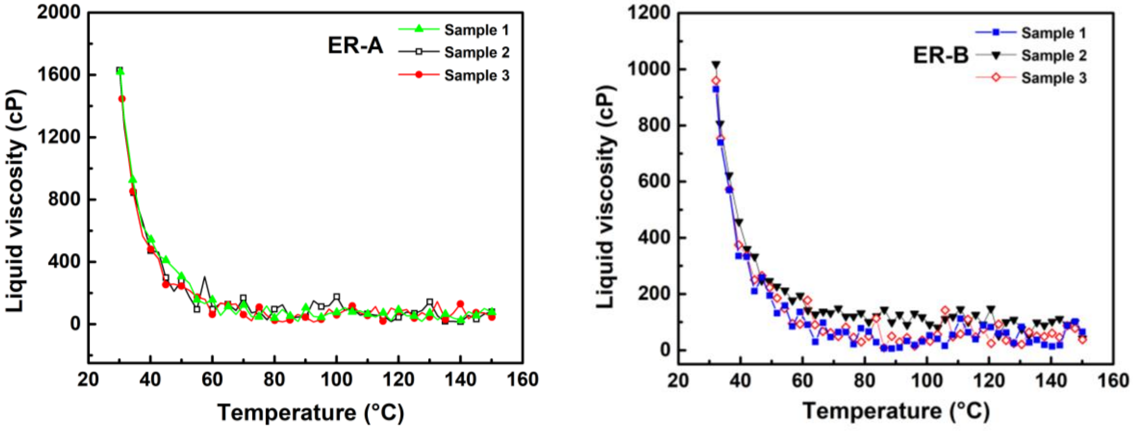

(**a**) (**b**)

Figure S1. Rheometry characterization of epoxy resins. (**a**) ER-A, (**b**) ER-B.

1.2. Thermoresistive Response of Samples Cured Using a Temperature Program

Figure S2 shows the representative thermoresistive response of a freestanding CNTY (without polymer) at the beginning of zone III, using an identical temperature program as that employed for specimens CNTY/ER-A. This analysis was conducted to isolate (and quantify) the inherent thermoresistive response of the yarn from the effect of resin pouring (interaction between the polymer and the CNTY). This is needed because the door of the oven where the molds are preheated needs to be open for 2–3 min, for resin pouring. As seen in Figure S2, the sole act of opening the oven's door for ~2–3 min yields a sharp decrease in temperature of ~ 100 °C (from ~ 128 to 28 °C), concomitant with a CNTY increase in electrical resistance (negative thermoresistive response), with $\Delta R/R_0$ changing from ~ −7.5% to ~ −5.5% (~ 2%). This value is significantly smaller than that experienced by CNTY/ER-A specimens in Figures 3a and 4c, and was considered as a subtracting term in the ERP parameter (Equation (3)) listed in Table 3. This means that the increase in *R* observed in Figure 4c is not only due to the thermoresistive effect of the CNTY, but there is a physicochemical interaction at the interphase between the CNTY and the polymer upon resin pouring. Wicking and infiltration into the external bundles of the CNTY are also expected [2].

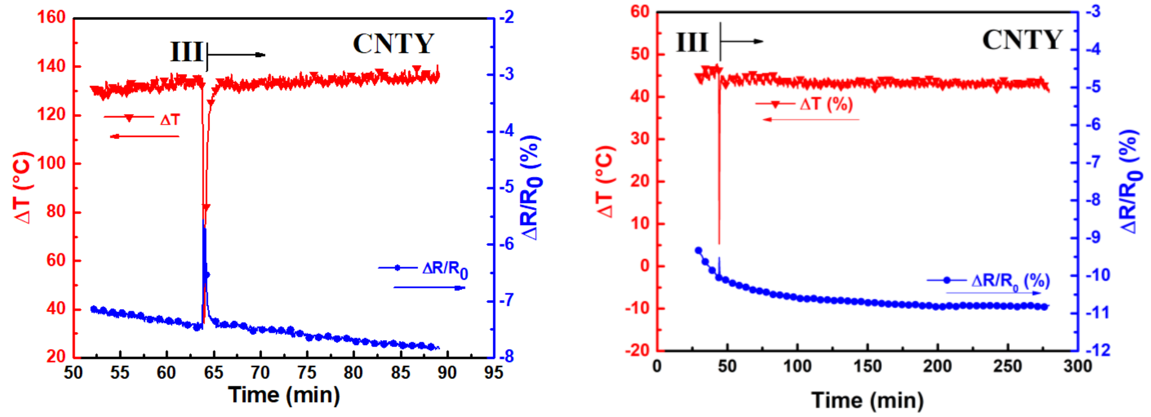

(**a**) (**b**)

Figure S2. Onset of zone III of freestanding CNTY using the same temperature program as for the CNTY/polymer experiment. (**a**) CNTY/ER-A, (**b**) CNTY/ER-B.

A similar situation is observed in Figure S2b for an individual CNTY (without polymer) at the beginning of zone III, mimicking the temperature program of samples CNTY/ER-B. In this case, the temperature decreases from 65 to 25 °C (~ 40 °C) upon opening the oven's door and $\Delta R/R_0$ changes from ~ −10.6% to ~ −9.8% (~ 0.8%). This is again much smaller than what CNTY/ER-B experiences in Figure 6b, and was accounted for in the calculation of ERP in Table 3.

1.3. Thermograms of Polymers Cured at Room Temperature

Figure S3 shows measurements of temperature over curing time for CNTY/ER-B, CNTY/ER-B-50 and only CNTY (without resin) specimens listed in Table 1, which were cured at RT. The experiments were conducted to examine potential changes in temperature (exotherms) during the polymerization process (or by Joule effect, in the case of Figure S3c), in order to account for or discard the contribution of this temperature effect on the electrical changes observed in the experiments. The measurements were conducted using a K-type thermocouple placed into the mold, i.e., embedded in the epoxy resin during curing for monofilament composite, or placed slightly above the CNTY, and covering the samples with a Petri dish. As seen from all Figure S3, negligible fluctuations in temperature were detected for these specimens. Thus, the changes showed in Figure 8 could not be attributed to exotherms changes in temperature, or Joule effect, but rather to physicochemical interactions between the polymer and the CNTY.

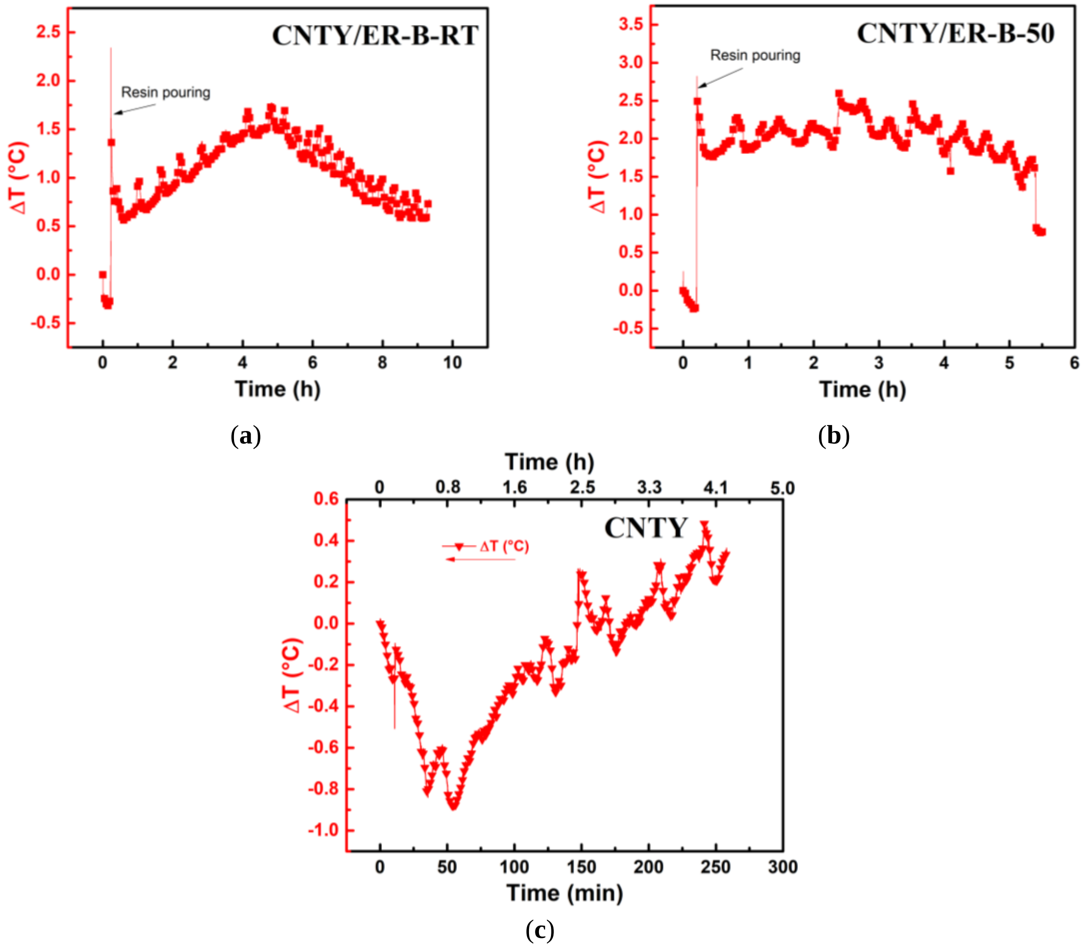

Figure S3. Thermograms of specimens cured at RT. (**a**) CNTY/ER-B-RT, (**b**) CNTY/ER-B-50, (**c**) only CNTY.

References

1. Jung, Y.; Kim, T.; Park, C.R. Effect of polymer infiltration on structure and properties of carbon nanotube yarns. *Carbon* **2015**, *88*, 60–69.
2. Sui, X.; Greenfeld, I.; Cohen, H.; Zhang, X.; Q. Li; Wagner, H.D. Multilevel composite using carbon nanotube fibers (CNTF). *Compos. Sci. Technol*. **2016**, *137*, 35–43.
